# Impact of Ambient Temperature on Mortality Burden and Spatial Heterogeneity in 16 Prefecture-Level Cities of a Low-Latitude Plateau Area in Yunnan Province: Time-Series Study

**DOI:** 10.2196/51883

**Published:** 2024-07-23

**Authors:** Yang Chen, Lidan Zhou, Yuanyi Zha, Yujin Wang, Kai Wang, Lvliang Lu, Pi Guo, Qingying Zhang

**Affiliations:** 1 School of Public Health, Kunming Medical University, Kunming, China; 2 Institute for Noncommunicable Disease Prevention and Control, Yunnan Centers for Disease Prevention and Control, Kunming, China; 3 Department of Preventive Medicine, Shantou University Medical College, Shantou, China; 4 Graduate School, Kunming University of Medical, Kunming, China

**Keywords:** mortality burden, nonaccidental deaths, multivariate meta-analysis, distributed lagged nonlinear mode, attributable risk, climate change, human health, association, temperature, mortality, nonaccidental death, spatial heterogeneity, meteorological data, temperature esposure, heterogeneous, spatial planning, environmental temperature, prefecture-level, resource allocation

## Abstract

**Background:**

The relation between climate change and human health has become one of the major worldwide public health issues. However, the evidence for low-latitude plateau regions is limited, where the climate is unique and diverse with a complex geography and topography.

**Objectives:**

This study aimed to evaluate the effect of ambient temperature on the mortality burden of nonaccidental deaths in Yunnan Province and to further explore its spatial heterogeneity among different regions.

**Methods:**

We collected mortality and meteorological data from all 129 counties in Yunnan Province from 2014 to 2020, and 16 prefecture-level cities were analyzed as units. A distributed lagged nonlinear model was used to estimate the effect of temperature exposure on years of life lost (YLL) for nonaccidental deaths in each prefecture-level city. The attributable fraction of YLL due to ambient temperature was calculated. A multivariate meta-analysis was used to obtain an overall aggregated estimate of effects, and spatial heterogeneity among 16 prefecture-level cities was evaluated by adjusting the city-specific geographical characteristics, demographic characteristics, economic factors, and health resources factors.

**Results:**

The temperature-YLL association was nonlinear and followed slide-shaped curves in all regions. The cumulative cold and heat effect estimates along lag 0‐21 days on YLL for nonaccidental deaths were 403.16 (95% empirical confidence interval [eCI] 148.14‐615.18) and 247.83 (95% eCI 45.73‐418.85), respectively. The attributable fraction for nonaccidental mortality due to daily mean temperature was 7.45% (95% eCI 3.73%‐10.38%). Cold temperature was responsible for most of the mortality burden (4.61%, 95% eCI 1.70‐7.04), whereas the burden due to heat was 2.84% (95% eCI 0.58‐4.83). The vulnerable subpopulations include male individuals, people aged <75 years, people with education below junior college level, farmers, nonmarried individuals, and ethnic minorities. In the cause-specific subgroup analysis, the total attributable fraction (%) for mean temperature was 13.97% (95% eCI 6.70‐14.02) for heart disease, 11.12% (95% eCI 2.52‐16.82) for respiratory disease, 10.85% (95% eCI 6.70‐14.02) for cardiovascular disease, and 10.13% (95% eCI 6.03‐13.18) for stroke. The attributable risk of cold effect for cardiovascular disease was higher than that for respiratory disease cause of death (9.71% vs 4.54%). Furthermore, we found 48.2% heterogeneity in the effect of mean temperature on YLL after considering the inherent characteristics of the 16 prefecture-level cities, with urbanization rate accounting for the highest proportion of heterogeneity (15.7%) among urban characteristics.

**Conclusions:**

This study suggests that the cold effect dominated the total effect of temperature on mortality burden in Yunnan Province, and its effect was heterogeneous among different regions, which provides a basis for spatial planning and health policy formulation for disease prevention.

## Introduction

The association between global climate change and human health has become a profound global public health problem [1-3]. It is marked by rising average surface temperatures and heightened weather instability [4,5]. Numerous epidemiological studies have shown that changes in ambient temperature are associated with increased risk of disease and mortality [6-10]. Both cold and heat exposure can have adverse effects on human health, including cause-specific mortality, emergency room visits, hospitalizations, and ambulance dispatch [11-15]. Specifically, temperature extremes are linked to an increased incidence of cardiovascular diseases, such as heart disease and stroke, as well as respiratory disease [16-18].

In recent years, numerous studies have investigated the impact of temperature on mortality, suggesting that nonoptimal temperature may elevate the risk of chronic diseases and infectious disease transmission, thereby increasing the overall mortality burden [19-21]. For example, a prominent investigation in this field includes a worldwide study that assessed the relative risk associated with nonoptimal temperatures on 176 specific causes of death [22]. The findings indicated distinct effects of heat and cold exposure on various causes of death. Additionally, a time-series analysis conducted in 272 major cities in China delved into the correlation between ambient temperature and years of life lost (YLL) for nonaccidental deaths, specifically focusing on cardiorespiratory disease [23]. The study identified an inverse J-shaped association between ambient temperature and the risk of mortality, highlighting the corresponding disease burden that is mainly attributable to moderate cold exposure in China. An association between ambient temperature and mortality risk has also been documented in India, the European Union, South Africa, and various other countries and regions [6,24-26].

However, the assessment of temperature-related mortality burden in these studies primarily involved the number of deaths or mortality rate as the prevailing indicators, treating all deaths equally important [6,7,27]. As compared with the number of deaths or mortality, YLL is more informative because it considers life expectancy and assigns a higher weight to deaths at younger ages [28]. Meanwhile, the attributable fraction (AF) can be combined with YLL to adequately reflect the effects of temperature exposure and ensure a comprehensive analysis. AF estimates the proportion of deaths that would not have occurred in the absence of an exposure, and it has important policy implications pointing to the potential impact of an intervention [29].

Despite numerous studies on the mortality burden of temperature, the results have been inconsistent due to global differences in environmental, geographical, economic, and demographic factors [8,30]. In other words, spatial heterogeneity should be considered when comparing different regions. However, existing studies on the spatial heterogeneity of the effect of temperature on mortality burden are limited, with a few focusing on specific causes of death or only on heat effects [4,17,31]. Moreover, comprehensively analyzing spatial heterogeneity is difficult when study areas are restricted to individual cities or study areas with little variability in geographic characteristics.

Yunnan Province is located on the Yunnan-Guizhou Plateau, with a particular geographical location, complex topography, and unique and diverse climatic environments. This situation results in a diversity of ethnic, economic, and health resource factors in Yunnan Province. Thus, the province is an ideal place to study the spatial heterogeneity of its different cities. Our previous studies examined the association between temperature exposure and YLL in several cities of Yunnan Province but did not investigate spatial heterogeneity because of lack of data from eastern, northern, and central cities [32,33].

Therefore, this study used the entire Yunnan Province counties, representative of the low-latitude plateau region, as the study area to focus on assessing the mortality burden due to mean temperature, populations sensitive to environmental temperature exposure, and high-risk disease causes of death. Additionally, the study aimed to explore the spatial heterogeneity of the impact of mean temperature on mortality burden. The findings may contribute to the development of early warning strategies aimed at mitigating the risk of additional mortality due to ambient temperature in the region. Furthermore, they can inform measures to protect vulnerable populations and guide the allocation of resources and policy planning for interventions.

## Methods

### Ethical Considerations

The data used for this analysis were aggregated and had no identifiable information about the study subjects. Therefore, this study did not require research ethics committee approval.

### Study Site

**Figure 1.**
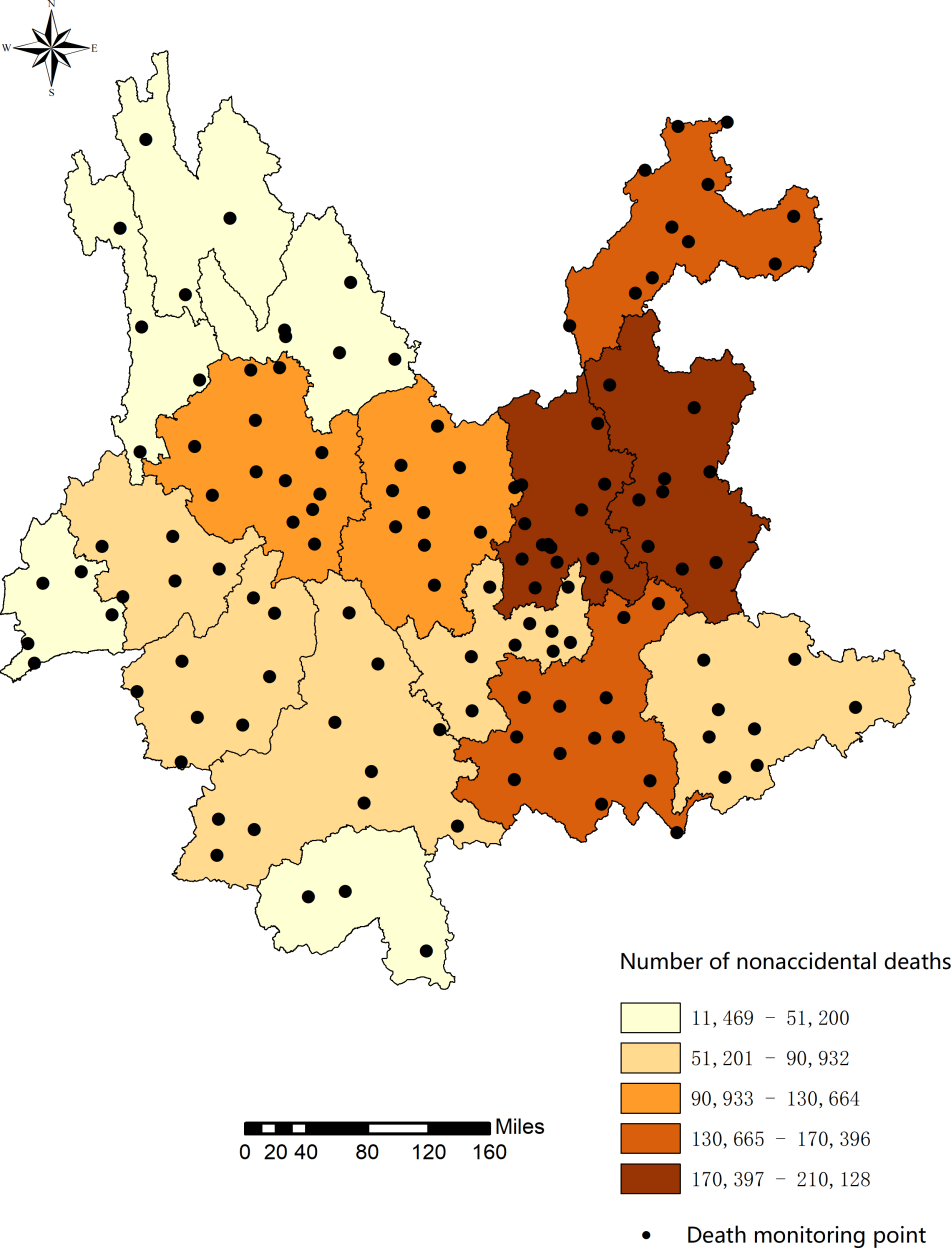
Spatial distribution of nonaccidental mortality for the 16 cities and 129 death monitoring points in Yunnan Province in China, from 2014 to 2020.

This study selected all 129 county-level mortality monitoring sites in Yunnan Province, Southwest China, using 16 prefecture-level cities as units of analysis (Figure 1). Yunnan Province belongs to the subtropical plateau monsoon climate zone, with remarkable 3D climate, various climate types, minor annual temperature differences, sizable daily temperature differences, and distinct wet and dry seasons. Additionally, the temperature changes vertically with terrain height.

### Data Collection

The study compiled all-cause mortality data for residents across all 129 counties in Yunnan Province from January 1, 2014, to December 31, 2020, using the Yunnan Centre for Disease Control and Prevention’s death registration reporting system. To define the 5 causes of death [23,34], we used codes from *the International Classification of Diseases, 10th Revision*, namely total nonaccidental deaths [A00–R99], cardiovascular disease [I00–I99], heart disease [I00–I51], stroke [I60–I69], and respiratory disease [J00–J99]. Furthermore, total nonaccidental deaths were classified according to age (<75 or ≥75 years), sex (male or female), occupation (farmer, staff, or other), ethnicity (Han nationality or ethnic minorities), marital status (married or nonmarried), and education level (junior college and above or below junior college).

Daily meteorological data for the corresponding period were acquired from the China Meteorological Data Sharing System, encompassing parameters such as mean temperature, wind speed, atmospheric pressure, relative humidity, and sunshine duration. The geographical and social characteristics of the 16 prefecture-level administrative regions during the same period were extracted from the China Statistical Yearbook and the Yunnan Statistical Yearbook. These characteristics encompassed geographical indicators (eg, latitude, longitude, and altitude), demographic indicators (eg, population density, natural population growth rate, and urbanization rate), economic indicators (eg, gross domestic product [GDP] per capita, GDP growth rate, and employment rate), and health resources (eg, the number of health technicians and hospital beds per 1000 population).

The Chinese national life expectancy table (Multimedia Appendix 1), accessible from the World Health Organization’s Global Health Observatory, offers age-specific life expectancy data for male and female individuals nationwide from 2010 to 2019. Drawing on prior research [35,36], life expectancy for the 2014‐2020 period was estimated by averaging the values from 2010 and 2019. The calculation of YLL for each death involved aligning age and sex with the reference life expectancy table, followed by the summation of YLL for all deaths on a given day to derive the daily YLL.

### Statistical Analysis

A 3-stage analysis method widely used in previous studies [37,38] was used to estimate the association between mean temperature and YLL. The district map of Yunnan Province was drawn with ArcGIS (version 10.8; Esri). All analyses were performed with R (version 4.1.0; R Foundation for Statistical Computing), using the packages “dlnm” and “mvmeta.” Two-sided *P*<.05 was considered statistically significant.

#### First Stage: Time-Series Analysis

In the first stage of the analysis, because of the normal distribution of daily YLL, we used a general linear regression model with a link Gaussian function combined with a distributed lag nonlinear model to assess the nonlinear and delayed effects of mean temperature on YLL [34,39,40]. Regarding the lag effect, according to previous studies, the maximum lag days was set to 21 days [9,38,41,42]. The general model is as follows:


$$YLL_{t}=\alpha +\beta Temp_{t,l}+NS\left(Time_{t},df=7\times 7\right)+NS\left(RH_{t},df=3\right)+NS\left(SD_{t},df=3\right)+NS\left(WS_{t},df=3\right)+NS\left(AP_{t},df=3\right)+\gamma DOW_{t}+\delta Holiday_{t}$$

where ${YLL}_{t}$ is the observed daily YLL at day *t* (*t*=1,2,3,...,2557), α is the intercept, and β is the regression coefficient for mean temperature. $Temp_{t,l}$ is the cross-base matrix of mean temperature and lagged effects generated by the distributed lag nonlinear model, with the 2 internal knots located at the 27.5th and 72.5th percentiles of the daily mean temperature distribution [43]; *l* is the maximum number of lagged days; NS() means a natural cubic spline; long-term trends and seasonality were controlled by a natural cubic B-spline with 7 *df* per year. Relative humidity (RH), wind speed (WS), sunshine duration (SD), and air pressure (AP) were controlled by a natural cubic spline with 3 *df* [38,44]. ${DOW}_{t}$ and ${Holiday}_{t}$ are days of the week and public holidays in China, represented as categorical variables included in the model; γ and δ are the regression coefficients for *DOW* and *Holiday*, respectively [38,45].

In this study, YLL estimates and their 95% empirical confidence intervals (eCIs) were calculated to assess the impact of temperature exposure on total nonaccidental mortality and its subgroups under the cold and heat effects. Cold and heat were defined as temperatures above and below the temperature with the lowest YLL.

#### Second Stage: Multivariate Meta-Analysis

In the second stage, city-specific estimates of the associations between temperature exposure and YLL obtained from the first stage were pooled by multivariate meta-regression. The analysis also focused on the cumulative exposure response association by summing lagged effects to avoid excessive model parameters and to improve statistical power [42,46].

A multivariate meta-regression was fitted using the intercept only to obtain an overall pooled estimate and to assess spatial heterogeneity. City-specific characteristics (eg, longitude, latitude, altitude, population density, natural population growth rate, urbanization rate, GDP per capita, GDP growth rate, employment rate, and number of health technicians and hospital beds per 1000 population) were separately included in the intercept-only models to determine whether specific meta-predictors could explain spatial heterogeneity. In addition, the Wald test was used to assess the significance of these individual meta-prediction models as compared with intercept-only models. All multivariate meta-regression models were estimated using maximum likelihood estimation, with residual heterogeneity tested by the Cochran *Q* test and the *I*^2^ statistic, and the goodness of fit of the models was measured using the Akaike Information Criterion [47].

#### Third Stage: Disease Burden Attributable to Ambient Temperature

A univariate random-effects meta-analysis was used to pool the risk estimates for each city to construct an overall cumulative exposure-response curve for all cities and to obtain the best linear unbiased prediction for each city [47,48]. The details of this method were previously described in previous studies [9,47]. Subsequently, we used the natural cubic spline function of the quadratic B-spline function governing the temperature-mortality association. The temperature with the lowest YLL of each city served as a reference point for calculating the estimated attributable risk for YLL. Finally, the AF was computed for both high- and low-temperature groups with the formula AF=attributable YLL / total YLL [29,49]. The 95% eCIs for AF were estimated by Monte Carlo simulation with 1000 resampling iterations [29]. The AF (%) with 95% eCIs was used to signify the impact of mean temperature on YLL.

## Results

### Data Description

The basic characteristics of the daily number of nonaccidental deaths in Yunnan Province from 2014 to 2020 are shown in Multimedia Appendix 2. The description results of daily YLL and weather conditions in Yunnan Province from 2014 to 2020 are shown in Table 1. The average daily YLL due to nonaccidental deaths was 546.1 person-years: 322.7 person-years were for men and 223.4 for women. Within the specific cause of death subgroups, the average daily YLL was 196.3 person-years for cardiovascular disease, 82.3 for respiratory disease, 87.7 for heart disease, and 98.6 for stroke. The daily mean temperature was 17.4 ℃, ranging from −4.8 ℃ to 29.3 ℃. The mean values for air pressure, relative humidity, wind speed, and sunshine hours were 844.4 hPa, 71.6%, 1.8 m/s, and 5.7 hours, respectively. The overall cumulative exposure-response curves (best linear unbiased prediction) for the 16 prefecture-level cities, as well as the distribution of temperatures, are shown in Multimedia Appendix 3.

**Table 1.** Descriptive statistics of daily years of life lost (YLL) and weather conditions in Yunnan Province, China, from 2014 to 2020.

Variable		Mean (SD)	Median (IQR)	Min, Max
Total nonaccidental mortality		546.1 (391.65)	473.7 (232.1-770.3)	0, 2969.3
**Cause-specific mortality**				
	Cardiovascular disease	196.3 (139.0)	177.7 (83.2-283.8)	0, 1081.7
	Heart disease	87.7 (71.6)	73.6 (31.4-128.4)	0, 604.9
	Stroke	98.6 (77.6)	86.0 (36.2-144.8)	0, 599.2
	Respiratory disease	82.3 (89.0)	54.7 (14.8-120.2)	0, 808.2
**Sex**				
	Male	322.7 (236.8)	275.9 (135.7-460.6)	0, 1782.4
	Female	223.4 (174.9)	186.4 (86.2-320.2)	0, 1393.5
**Age (years)**				
	<75	436.5 (310.2)	376.9 (192.0-619.1)	0, 2393.3
	≥75	109.5 (93.7)	88.6 (33.9-157.2)	0, 765.3
**Ethnicity**				
	Han nationality	384.6 (372.0)	293.4 (100.4-506.9)	0, 2884.9
	Minorities	160.2 (137.6)	123.7 (57.9-223.1)	0, 1079.7
**Marital status**				
	Married	467.8 (338.4)	407.8 (193.8-665.7)	0, 2747.0
	Nonmarried	78.2 (87.7)	59.7 (0-119.1)	0, 841.4
**Occupation**				
	Farmer	416.3 (297.4)	380.9 (167.4-588.6)	0, 2659.3
	Staff	20.2 (83.8)	7.3 (0-16.2)	0, 775.5
	Other	109.5 (356.2)	64.6 (0-147.7)	0, 1836.5
**Education level**				
	Junior college and above	11.2 (24.1)	0 (0-11.3)	0, 279.5
	Below junior college	534.9 (382.5)	466.2 (227.4-757.2)	0, 2889.1
**Daily meteorological measures**				
	Mean temperature (°C)	17.4 (5.6)	18.4 (13.6-22.0)	−4.8, 29.3
	Air pressure (hPa)	844.4 (47.7)	850.5 (818.0-878.1)	704.3, 915.5
	Relative humidity (%)	71.6 (13.6)	74.0 (63.0-82.0)	21.0, 99.0
	Wind speed (m/s)	1.8 (0.8)	1.6 (1.2-2.2)	0.3, 5.5
	Sunshine duration (h)	5.7 (3.3)	6.2 (2.9-8.6)	0, 12.4

**Figure 2.**
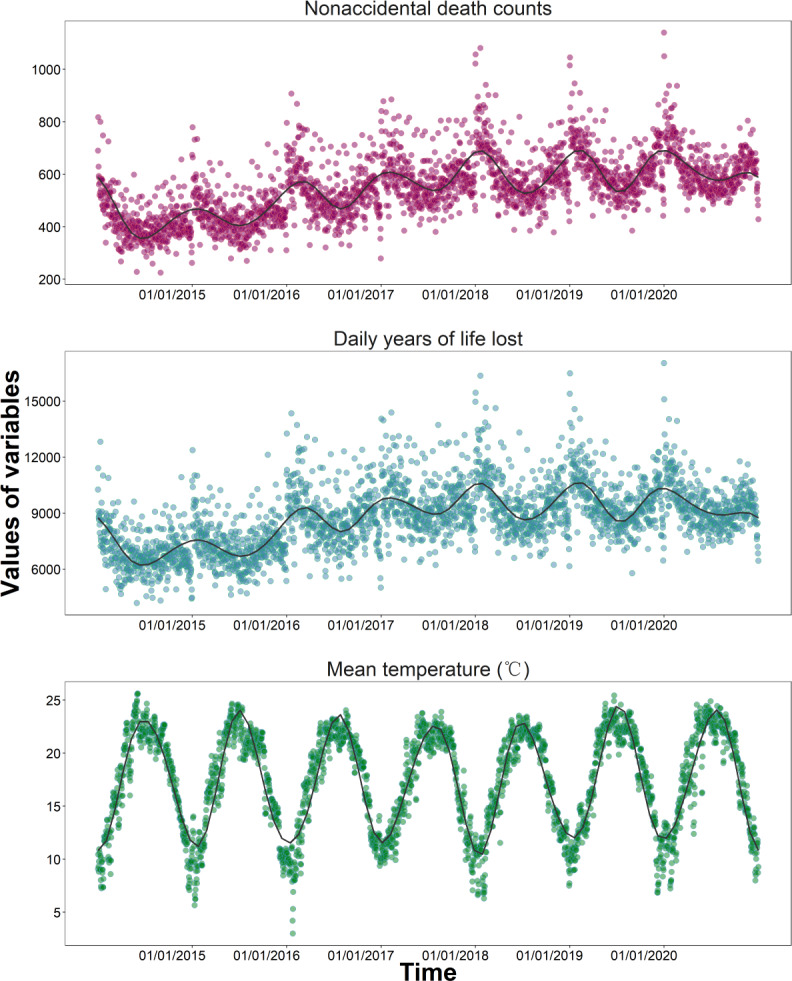
Time series of daily number of nonaccidental deaths, daily years of life lost, and daily mean temperature from 2014 to 2020.

Figure 2 depicts the time series curves for the number of nonaccidental deaths, YLL per day, and daily mean temperature for the 2014‐2020 period. the average daily nonaccidental deaths and daily life expectancy loss show a gradual annual increase, with relatively high values in January and December. There was a clear seasonality in the daily mean temperature, with relatively constant peaks each year—low peaks in January and December and from June to August.

### Impact of Daily Mean Temperature on YLL

#### Association Between Temperature and YLL

**Figure 3.**
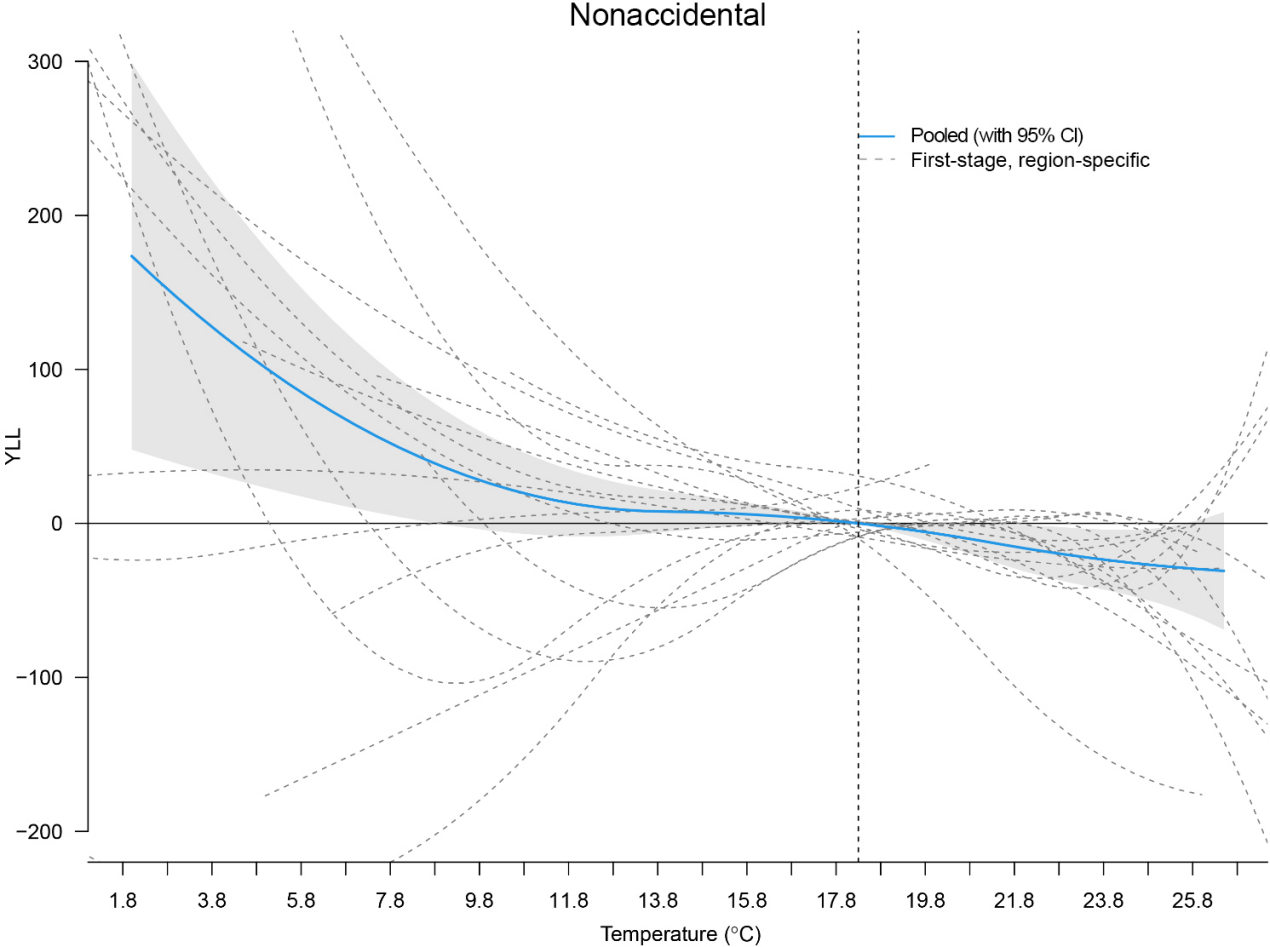
The dose-response curves for mean temperature and years of life lost (YLL) due to nonaccidental mortality.

Figure 3 shows a plot of the overall cumulative effect of the daily mean temperature of YLL for nonaccidental deaths for the 16 cities and a plot of the cumulative effect aggregated for Yunnan Province. The values marked by dotted lines in the graph are the 50th percentile of the daily mean temperature, and YLL values were calculated for different mean temperature levels relative to the 50th percentile. The association between daily mean temperature and YLL was nonlinear and had a “sliding scale” shape. Table 2 shows the YLL estimates for the effect of different temperature levels on nonaccidental deaths and their subgroups. Overall, the effect of temperature exposure, especially cold, on the YLL for nonaccidental deaths was significant, with 21 -day lagged effect estimates of YLL for nonaccidental deaths of 650.99 (95% eCI 325.92‐906.57) for the overall effect and 403.16 (95% eCI 148.14‐615.18) for the cold effect. The YLL values for the cardiovascular disease subgroup were significantly increased under cold conditions (304.90, 95% eCI 175.35‐405.62), with no significant effect under heat conditions (35.90, 95% eCI −11.75 to 73.50). Among male individuals, a noteworthy YLL estimate was observed under cold conditions (302.93, 95% eCI 187.41-391.77) as compared with 167.64 (95% eCI −2.34 to 307.37) under heat conditions. In the age group <75 years, under cold conditions, the YLL estimate was 403.63 (95% eCI 162.95‐582.02), and for those aged ≥75 years, it was 65.81 (95% eCI 32.92‐93.17). Additionally, for married individuals, under cold conditions, the YLL estimate was 313.41 (95% eCI 141.19‐458.99) in contrast to 66.44 (95% eCI 1.66‐107.83) for nonmarried individuals under the same conditions.

**Table 2.** Estimated daily years of life lost (YLL) for specific subgroups at different temperature levels reported with 95% empirical confidence intervals (eCIs).

Predictors		Overall effect, YLL (95% eCI)	Cold^a^ effect, YLL (95% eCI)	Heat^a^ effect, YLL (95% eCI)
Total nonaccidental mortality		650.99 (325.92 to 906.57)	403.16 (148.14 to 615.18)	247.83 (45.73 to 418.85)
**Cause-specific mortality**				
	Cardiovascular disease	340.80 (210.46 to 440.16)	304.90 (175.35 to 405.62)	35.90 (−11.75 to 73.50)
	Heart disease	195.99 (86.89 to 274.14)	174.10 (75.55 to 236.96)	21.89 (−13.86 to 46.53)
	Stroke	159.79 (95.12 to 207.88)	129.90 (77.08 to 168.21)	29.90 (−0.37 to 56.13)
	Respiratory disease	146.48 (33.14 to 221.59)	59.85 (13.89 to 95.40)	86.63 (−8.63 to 149.76)
**Sex**				
	Male	470.57 (255.36 to 640.04)	302.93 (187.41 to 391.77)	167.64 (−2.34 to 307.37)
	Female	258.98 (115.69 to 368.94)	150.48 (34.81 to 245.90)	108.50 (44.85 to 164.79)
**Age (years**)				
	<75	635.36 (353.17 to 874.06)	403.63 (162.95 to 582.02)	231.73 (51.74 to 377.31)
	≥75	87.64 (37.64 to 130.92)	65.81 (32.92 to 93.17)	21.84 (−18.78 to 57.09)
**Ethnicity**				
	Han nationality	405.94 (96.07 to 666.61)	200.02 (−22.52 to 370.54)	205.92 (−32.86 to 392.39)
	Minorities	264.17 (145.78 to 347.78)	214.21 (115.72 to 294.22)	49.96 (−3.56 to 95.14)
**Marital status**				
	Married	549.74 (283.55 to 762.00)	313.41 (141.19 to 458.99)	236.33 (48.97 to 398.86)
	Nonmarried	140.07 (32.51 to 205.71)	66.44 (1.66 to 107.83)	73.63 (−8.52 to 130.60)
**Occupation**				
	Farmer	392.95 (169.37 to 528.59)	200.60 (120.24 to 258.14)	189.14 (3.10 to 309.40)
	Staff	72.48 (21.23 to 92.07)	45.20 (6.57 to 63.42)	27.77 (2.58 to 38.85)
	Other	228.57 (114.40 to 284.49)	144.21 (66.05 to 190.19)	86.10 (3.66 to 124.02)
**Education level**				
	Junior college and above	38.84 (−2.92 to 52.17)	22.09 (3.92 to 26.19)	16.75 (−17.78 to 35.52)
	Below junior college	635.13 (337.53 to 885.24)	391.94 (145.06 to 604.87)	243.19 (46.45 to 403.71)

a Cold and heat were defined as temperatures below and above the temperatures with the lowest YLL.

#### Spatial Heterogeneity of the Effect of Mean Temperature on YLL

The results of the meta-analysis (intercept only) and the multivariate meta-regression (1 meta-predictor) of the mean temperature with YLL random effects are shown in Table 3. In the analysis of spatial heterogeneity in the effect of mean temperature on YLL, the heterogeneity across cities was statistically significant according to Cochran’s *Q* test (*Q*=115.8; *P*<.001), with 48.2% of the heterogeneity due to actual differences between the 16 prefecture cities. Urban characteristics, such as longitude, population density, urbanization rate, GDP per capita, and the number of health technicians and hospital beds per 1000 population, could explain some of the spatial heterogeneity, with urbanization rate (15.7%) accounting for the highest proportion of heterogeneity.

**Table 3.** Spatial heterogeneity of the effect of mean temperature on years of life lost.

Predictors		Wald test			Model fit	Cochran *Q* test			*I* ^2^
		*W* value	*df*	*P* value	AIC^a^	*Q* value	*df*	*P* value	(%)
Reference model (intercept)		-	-	-	792.0	115.8	60	<.001	48.2
**Geographical indicators**									
	Longitude	21.9	4	<.001	755.4	87.6	56	.004	36.1
	Latitude	2.9	4	.57	767.2	109.3	56	<.001	48.8
	Altitude	4.4	4	.36	814.9	105.1	56	<.001	46.7
**Demographic indicators**									
	Population density	19.6	4	<.001	790.5	90.0	56	.003	37.8
	Natural population growth rate	2.8	4	.59	765.8	111.1	56	<.001	49.6
	Urbanization rate	31.6	4	<.001	732.6	83.0	56	.01	32.5
**Economic indicators**									
	GDP^b^ per capita	13.9	4	.007	826.9	100.5	56	<.001	44.3
	GDP growth rate	4.4	4	.36	735.7	102.2	56	<.001	45.2
	Employment rate	7.9	4	.10	733.5	108.2	56	<.001	48.3
**Health resource indicators**									
	Number of health technicians per 1000 population	11.1	4	.03	758.3	105.3	56	<.001	46.8
	Number of hospital beds per 1000 population	14.6	4	.005	755.6	93.3	56	.001	40.0

a AIC: Akaike Information Criterion.

b GDP: gross domestic product.

#### Attributable Risk for the Effect of Mean Temperature on YLL

The impact of mean temperature on the total AF for nonaccidental deaths and their subgroups is presented in Table 4. Overall, the total proportion of deaths due to temperature was 7.45% (95% eCI 3.73‐10.38). Cold effect explained most of the attributable risk (4.61%, 95% eCI 1.70‐7.04) compared with heat effect (2.84%, 95% eCI 0.58‐4.83). In the cause-specific subgroup analysis, the total AF (%) for mean temperature was 13.97% (95% eCI 6.70‐14.02) for heart disease, 11.12% (95% eCI 2.52‐16.82) for respiratory disease, 10.85% (95% eCI 6.70‐14.02) for cardiovascular disease, and 10.13% (95% eCI 6.03‐13.18) for stroke. The attributable risk of cold effect was higher for cardiovascular than respiratory disease cause of death (9.71% vs 4.54%). In the subgroup analysis of individual characteristics, the total effect of daily mean temperature on mortality was 9.11% (95% eCI 4.95‐12.40) for male individuals, 9.10% (95% eCI 5.06‐12.51) for people aged <75 years, 10.30% (95% eCI 5.69‐13.57) for minorities, 11.19% (95% eCI 2.60‐16.44) for nonmarried individuals, 17.74% (95% eCI 7.64‐23.86) for farmers, and 7.42% (95% eCI 3.94‐10.34) for those with education below junior college level, higher than their corresponding counterparts. Except for the Han ethnicity group, the cold effect was similar to the overall effect of temperature exposure, and the effects were higher for populations mentioned above compared to their corresponding counterparts.

**Table 4.** Attributable fraction (%) of years of life lost to total nonaccidental mortality due to mean temperature and the specified subgroups. Attributable fractions (%) are reported with 95% empirical confidence intervals (eCIs)

Predictors		Overall effect, % (95% eCI)	Cold^a^ effect, % (95% eCI)	Heat^a^ effect, % (95% eCI)
Total nonaccidental mortality		7.45 (3.73 to 10.38)	4.61 (1.70 to 7.04)	2.84 (0.58 to 4.83)
**Cause-specific mortality**				
	Cardiovascular disease	10.85 (6.70 to 14.02)	9.71 (5.58 to 12.92)	1.14 (−0.37 to 2.34)
	Heart disease	13.97 (6.19, to 19.54)	12.41 (5.39 to 16.89)	1.56 (−0.99 to 3.32)
	Stroke	10.13 (6.03 to 13.18)	8.23 (4.89 to 10.66)	1.90 (−0.02 to 3.56)
	Respiratory disease	11.12 (2.52 to 16.82)	4.54 (1.05 to 7.24)	6.58 (−0.66 to 11.37)
**Sex**				
	Male	9.11 (4.95 to 12.40)	5.87 (3.63 to 7.59)	3.25 −0.05 to 5.95)
	Female	7.25 (3.24 to 10.32)	4.21 (0.97 to 6.88)	3.04 (1.25 to 4.61)
**Age (years**)				
	<75	9.10 (5.06 to 12.51)	5.78 (2.33 to 8.33)	3.32 (0.74 to 5.40)
	≥75	5.00 (2.15 to 7.47)	3.76 (1.88 to 5.32)	1.25 (−1.07 to 3.26)
**Ethnicity**				
	Han nationality	6.60 (1.56 to 10.83)	3.25 (−0.37 to 6.02)	3.35 (−0.53 to 6.38)
	Minorities	10.30 (5.69 to 13.57)	8.36 (4.51 to 11.48)	1.95 (−0.14 to 3.71)
**Marital status**				
	Married	7.34 (3.79 to 10.18)	4.19 (1.89 to 6.13)	3.16 (0.65 to 5.33)
	Nonmarried	11.19 (2.60 to 16.44)	5.31 (0.13 to 8.62)	5.88 (−0.68 to 10.43)
**Occupation**				
	Farmer	17.74 (7.64 to 23.86)	8.51 (5.10 to 10.95)	9.53 (−0.79 to 16.26)
	Staff	12.34 (3.62 to 15.68)	7.23 (1.05 to 10.15)	5.11 (−5.80 to 11.5)
	Other	14.38 (7.20 to 17.9)	8.52 (3.90 to 11.24)	5.86 (−0.74 to 9.29)
**Education level**				
	Junior college and above	21.67 (−1.63 to 29.11)	12.33 (2.19 to 14.61)	9.35 (−9.92 to 19.82)
	Below junior college	7.42 (3.94 to 10.34)	4.58 (1.70 to 7.07)	2.84 (0.54 to 4.72)

a Cold and heat were defined as temperatures below and above the temperatures with the lowest YLL.

### Sensitivity Analysis

We conducted sensitivity analyses to assess the robustness of the specified parameters in the model. Variations, such as changing the maximum lag to 14 or 28 days and adjusting the degrees of freedom for the time variable to 6/year or 8/year as well as for the meteorological variable to 2 or 4, did not yield a significant difference in the AF related to YLL for nonaccidental deaths concerning the temperature exposure indicator (Multimedia Appendix 4). Thus, the model demonstrated relative robustness.

## Discussion

### Principal Findings

Using data from 16 prefecture-level cities of Yunnan Province, China, this province-wide research assessed the mortality burden attributable to temperature and further estimated its spatial heterogeneity. In general, the cumulative effects of ambient temperature on YLL for nonaccidental deaths were nonlinear, with cold exposure contributing significantly to YLL. Moreover, geographic factors, sociodemographic information, economic status, and health resources were potential causes of spatial heterogeneity in the effect of ambient temperature on the mortality burden.

Our findings showed that temperature was responsible for a significant proportion of YLL, corresponding to 7.45% of the mortality burden during the study period. As compared with high temperature, most of the mortality burden was due to exposure to low temperature (4.61% vs 2.84%), consistent with findings from previous multicity mortality studies [9,34,36,38]. Moreover, in the association between mean temperature and YLL, the effect estimates were higher for cold exposure than heat. The estimate of the YLL fraction attributable to ambient temperature was much lower than that reported in a previous study in Shenzhen (17.28%) [34]. This discrepancy may be explained by the special geographic location and climate of Yunnan province. Yunnan Province had a distinct subtropical plateau monsoon climate, displaying a low seasonal variation in temperature. Hence, temperature may have less effect on residents of Yunnan Province than those living in other areas. Additionally, improving the health monitoring system to promptly observe temperature variations and monitor the population’s health status is recommended. This facilitates early anomaly detection, enabling timely implementation of preventive and therapeutic interventions.

Several studies have investigated the mortality burden of temperature on different causes of death [22,23,50,51]. By comparing the AF and YLL effect values of temperature on different causes of death, we found that the effects of temperature, especially cold, were more pronounced for cardiovascular than respiratory disease, which is consistent with previous studies [35,52]. The effect of cold exposure on cardiovascular system is usually due to potential complications associated with increased cardiovascular risk related to changes in the autonomic nervous system, blood pressure, inflammatory response, and oxidative stress [53,54]. The effect of cold exposure on the respiratory system might be due to increased respiratory infections in cold days [55]. In cardiovascular disease, the increase in mortality burden from heart disease and stroke was associated with ambient air temperature, and the attributable risk and YLL effect estimates for mean air temperature were higher for heart disease compared to stroke.

In comparison with prior studies conducted in Yunnan Province, our research revealed that cardiovascular disease was also affected by daily temperature changes [32]. This discovery contributes to a more nuanced understanding of the interplay between meteorological factors and cardiovascular as well as cerebrovascular disease, offering valuable insights for preventing and managing these health issues. Diverging from the findings of other investigations, we found that the influence of heat exposure on cardiovascular and respiratory disease lacked statistical significance. This discrepancy may be attributed to the distinctive temperature distribution in Yunnan, where the maximum temperature reached only 29.3 °C during the study period. In contrast, national and Shenzhen-based time series studies, with maximum temperatures reaching 35.6 °C and 33 °C, respectively, confirm the significant impact of heat exposure on these diseases [34,50]. Future research should consider a broader temperature gradient to ensure more comprehensive and comparable results. According to the study findings, it is advisable to disseminate information regarding the health effects of temperature fluctuations and educate the public on adopting suitable precautionary measures, particularly during extreme temperatures, to mitigate health risks. Moreover, improving the health monitoring system to promptly observe temperature variations and monitor the population’s health status is recommended. This facilitates early anomaly detection, enabling timely implementation of preventive and therapeutic interventions.

Numerous studies have shown that demographic characteristics are essential modifiers of the association between mean temperature and health [56,57]. In our study, we analyzed the modifying effects of potential confounders, such as sex, age, ethnic group, and occupation, on the effect of identifying susceptible populations to temperature. The mortality burden of temperature was greater for male individuals, people <75 years of age, nonmarried individuals, farmers, and those with an education level below junior college level compared to their corresponding counterparts. Previous research has substantiated that men are susceptible to the effects of cold [34]. This difference in the effect of temperature exposure on the mortality burden by sex may be due to biological differences and socioeconomic factors. Consistent with our findings, numerous studies have identified a higher risk of mortality associated with cold exposure in younger versus older age groups [34,57]. The observed outcome can be attributed to the prevalent practice among most older adults of staying indoors during the cold season to mitigate direct exposure to temperature extremes. Consequently, the difference in the extent and duration of exposure between younger and older age groups is the primary factor contributing to this divergence. We found that ethnic minorities, nonmarried individuals, and farmers were more sensitive to the effects of temperature exposure. Only a few previous studies have analyzed the modifying effects of ethnic group, marital status, and occupation on temperature effects [16,58,59]. Each ethnic group has its culture, food habits, and way of life, so ethnicity is also an essential factor. Compared to married individuals, those who are unmarried tend to engage more in social activities and take part in more outdoor sports. Compared to “staff” or “others,” farmers primarily work outdoors, which exposes them more to ambient temperature and makes them more sensitive to climate change compared to nonfarming populations. Therefore, protective measures are needed to reduce the adverse effects of climate change for outdoors. Additionally, education level is widely recognized as a key indicator of socioeconomic status. Individuals with lower levels of education often experience poorer living conditions and health statuses, engage in riskier behaviors, and have limited access to health care services [36].

This study considered geographical factors, sociodemographic information, economic status, and health resources as potential modifying factors and found spatial heterogeneity in the effect of temperature on the mortality burden across regions. The highest percentage of spatial heterogeneity was due to the urbanization rate. An early study in Zhejiang Province, China, found a rural mortality burden due to cold exposure of up to 16.4%, much higher than the urban estimate of 7.0% [60]. This finding may be due to the urban heat island effect, with urban temperatures noticeably higher than rural temperatures. GDP per capita was this study’s most crucial correction factor. A study of 272 major cities in China found that GDP per capita and urbanization rates could alter the temperature-YLL association and its lag structure [23]. Another study showed a robust adverse effect of temperature on mortality in Asian cities with low GDP, with no significant association observed in cities with a higher GDP [61]. We found that health resources (ie, health technicians per 1000 population and hospital beds per 1000 population) similarly explained some of the heterogeneity in the temperature-YLL association. Areas with adequate health resources have better access to health care for their residents. Differences due to such health resource effects are more difficult to explain because evidence from relevant studies is sparse worldwide. Further epidemiological investigation is needed to elucidate whether and how health resources moderate or change the attributable risk of temperature on mortality.

A significant proportion of the spatial heterogeneity in the effect of mean temperature on YLL could also be attributed to longitude and population density. Available epidemiological evidence suggests that latitude and longitude may modify the association between temperature and mortality [62]. Because geographical coordinates, such as longitude, are usually highly correlated with local climatic characteristics (especially temperature distribution), the modification effect of geographical characteristics can be considered an indirect contribution to climate adaptation [63]. The sensitivity to temperature varies among individuals residing at different latitudes and longitudes. Areas with high population density primarily consist of economically developed cities, urban regions with industrial activities and vehicular emissions contributing to heat exposure, with the combined impact of heat island effects resulting from residential energy consumption and urban building structures. These factors, along with influences on health resources, environmental conditions, and others, can affect the association between temperature and population health [64].

### Strengths and Limitations

There are some strengths and weaknesses to our study. The strengths include the fact that it was a comprehensive and systematic multicity study that quantified the impact of temperature exposure on the burden of mortality in Yunnan Province, southwestern China. In addition, it was the first study in Yunnan Province to explore the causes of potential heterogeneity from multiple perspectives, including geography, socioeconomic status, and health sources. However, the study had some limitations. First, the results apply only to Yunnan Province, so caution should be exercised when generalizing its findings to other, particularly nonhighland, geographical areas. Second, the study assumed that everyone was exposed to the same mean temperature simultaneously, so there may be some exposure measurement bias. Third, a proportion of the heterogeneity in the results remains unexplained. Because of lack of available data, there may be essential correction factors that we failed to consider, such as vegetation cover, soil characteristics, and public health interventions.

### Conclusions

In summary, temperature exposure seems to exert a substantial impact on population health across the 16 prefecture-level administrative regions in Yunnan, particularly affecting individuals with cardiovascular and respiratory diseases. The cumulative effects of ambient temperature on YLL for nonaccidental deaths showed a nonlinear pattern, with cold exposure contributing more significantly than heat exposure to this effect. Specifically, under cold conditions, male individuals, nonmarried individuals, ethnic minorities, and farmers were more susceptible. In addition, after considering the effects of geographic characteristics, sociodemographic information, economic status, and health resources, we observed moderate spatial heterogeneity among the 16 prefecture-level cities in Yunnan Province. The implications of our study extend to both the research domain and the public health policy arena. The insights gained may assist policy makers in formulating effective intervention strategies, fortifying protection measures for vulnerable populations, and forecasting the potential impacts of climate change in this specific region.

## Supplementary material

10.2196/51883 Multimedia Appendix 1 Life expectancy for the Chinese population.

10.2196/51883 Multimedia Appendix 2 Summary statistics for daily nonaccidental mortality by mortality causes and individual characteristics in Yunnan Province, China, from 2014 to 2020.

10.2196/51883 Multimedia Appendix 3 Cumulative effect of temperature on nonaccidental deaths and its temperature distribution in 16 prefecture-level cities.

10.2196/51883 Multimedia Appendix 4 Sensitivity analysis.

## Abbreviations

AF: attributable fraction
eCI: empirical confidence interval
GDP: gross domestic product
YLL: years of life lost
