# Supplementary material for: Impact of Ambient Temperature on Mortality Burden and Spatial Heterogeneity in 16 Prefecture-Level Cities of a Low-Latitude Plateau Area in Yunnan Province: Time-Series Study
Source: JMIR Public Health Surveill. 2024 Jul 23;10:e51883. doi: 10.2196/51883 (PMC11287102; doi:10.2196/51883)
Supplement: Multimedia Appendix 1 [file publichealth-v10-e51883-s001.docx]

Table S1 Life expectancy table for Chinese population

| Age (years) | 2010 | |  | 2015 | |  | 2019 | |  | Mean | |
| --- | --- | --- | --- | --- | --- | --- | --- | --- | --- | --- | --- |
|  | Male | Female |  | Male | Female |  | Male | Female |  | Male | Female |
| 0-1 | 72.3 | 77.9 |  | 73.9 | 79.9 |  | 74.7 | 80.5 |  | 73.6 | 79.4 |
| 1-4 | 72.3 | 77.9 |  | 73.6 | 79.6 |  | 74.3 | 80.0 |  | 73.4 | 79.2 |
| 5-9 | 68.5 | 74.1 |  | 69.7 | 75.7 |  | 70.4 | 76.1 |  | 69.5 | 75.3 |
| 10-14 | 63.6 | 69.2 |  | 64.8 | 70.7 |  | 65.4 | 71.2 |  | 64.6 | 70.4 |
| 15-19 | 58.7 | 64.2 |  | 59.9 | 65.8 |  | 60.5 | 66.2 |  | 59.7 | 65.4 |
| 20-24 | 53.8 | 59.3 |  | 55.0 | 60.9 |  | 55.6 | 61.3 |  | 54.8 | 60.5 |
| 25-29 | 49.1 | 54.4 |  | 50.2 | 56.0 |  | 50.9 | 56.4 |  | 50.0 | 55.6 |
| 30-34 | 44.3 | 49.6 |  | 45.4 | 51.1 |  | 46.1 | 51.4 |  | 45.3 | 50.7 |
| 35-39 | 39.6 | 44.7 |  | 40.7 | 46.2 |  | 41.3 | 46.6 |  | 40.6 | 45.8 |
| 40-44 | 35.0 | 39.9 |  | 36.0 | 41.3 |  | 36.7 | 41.7 |  | 35.9 | 41.0 |
| 45-49 | 30.5 | 35.2 |  | 31.5 | 36.5 |  | 32.1 | 36.9 |  | 31.4 | 36.2 |
| 50-54 | 26.2 | 30.5 |  | 27.0 | 31.8 |  | 27.6 | 32.2 |  | 26.9 | 31.5 |
| 55-59 | 21.9 | 25.9 |  | 22.7 | 27.2 |  | 23.3 | 27.6 |  | 22.7 | 26.9 |
| 60-64 | 17.9 | 21.5 |  | 18.7 | 22.7 |  | 19.2 | 23.1 |  | 18.6 | 22.4 |
| 65-69 | 14.2 | 17.4 |  | 14.9 | 18.5 |  | 15.3 | 18.8 |  | 14.8 | 18.2 |
| 70-74 | 10.8 | 13.5 |  | 11.4 | 14.5 |  | 11.7 | 14.8 |  | 11.3 | 14.3 |
| 75-79 | 7.8 | 10.1 |  | 8.3 | 11.0 |  | 8.7 | 11.2 |  | 8.3 | 10.8 |
| 80-84 | 5.3 | 7.2 |  | 5.7 | 7.9 |  | 5.9 | 8.1 |  | 5.6 | 7.7 |
| 85+ | 3.2 | 5.0 |  | 3.3 | 5.5 |  | 3.7 | 5.0 |  | 3.4 | 5.2 |
