# Supplementary material for: Impact of Ambient Temperature on Mortality Burden and Spatial Heterogeneity in 16 Prefecture-Level Cities of a Low-Latitude Plateau Area in Yunnan Province: Time-Series Study
Source: JMIR Public Health Surveill. 2024 Jul 23;10:e51883. doi: 10.2196/51883 (PMC11287102; doi:10.2196/51883)
Supplement: Multimedia Appendix 2 [file publichealth-v10-e51883-s002.docx]

Table S2 Summary statistics for daily non-accidental mortality, by mortality causes and individual characteristics, in Yunnan Province, China, 2014–2020 (n=1401556)

|  | **Total deaths (%)** | **Mean (SD)** | **Median (Q1, Q3)** | **Min, Max** |
| --- | --- | --- | --- | --- |
| **Total non-accidental mortality** | 1,401,556 (100.00%) | 34.3 (26.02) | 30 (13 ,48) | 0, 207 |
| **Cause-specific** | | | | |
| Cardiovascular disease | 584,441 (41.70%) | 14.3 (10.22) | 13 (6 ,21) | 0, 85 |
| Heart disease | 284,206 (48.63%) | 6.9 (5.34) | 6 (3 ,10) | 0, 52 |
| Stroke | 293,397 (50.20%) | 7.2 (5.66) | 6 (3 ,11) | 0, 46 |
| Respiratory disease | 299,922 (21.40%) | 7.3 (7.95) | 5 (1 ,11) | 0, 74 |
| **Sex** | | | | |
| Male | 820,095 (58.51%) | 20.0 (15.47) | 17 (8 ,28) | 0, 126 |
| Female | 581,461 (41.49%) | 14.2 (11.25) | 12 (5 ,20) | 0, 86 |
| **Age (years)** | | | | |
| <75 | 735,011 (52.44%) | 18.0 (12.76) | 16 (8 ,26) | 0, 99 |
| $\geq$75 | 666,545 (47.56%) | 16.3 (14.16) | 13 (5 ,23) | 0, 118 |
| **Ethnic** | | | | |
| Han nationality | 1,033,417 (73.73%) | 25.3 (24.77) | 20 (6 ,33) | 0, 201 |
| Minorities | 365,598 (26.09%) | 8.9 (7.53) | 7 (3 ,12) | 0, 64 |
| **Marital status** | | | | |
| Married | 1,331,708 (95.02%) | 32.6 (24.97) | 28 (12 ,46) | 0, 200 |
| Non-married | 69,848 (4.98%) | 1.7 (1.73) | 1 (0 ,3) | 0, 15 |
| **Occupation** | | | | |
| Farmer | 1,128,161 (80.49%) | 27.6 (20.59) | 25 (10 ,40) | 0, 187 |
| Non-farmer | 273,395 (19.51%) | 6.7 (9.09) | 4 (2 ,7) | 0, 86 |
| **Education attainment** | | | | |
| Junior college and above | 22,588 (1.61%) | 0.6 (1.20) | 0 (0 ,1) | 0, 13 |
| Below junior college | 1,378,968 (98.39%) | 33.7 (25.45) | 29 (13 ,48) | 0, 205 |

Q1 = upper quartile; Q3 = lower quartile; Min = minimum; Max = max
