# Supplementary figures and images for: Impact of Ambient Temperature on Mortality Burden and Spatial Heterogeneity in 16 Prefecture-Level Cities of a Low-Latitude Plateau Area in Yunnan Province: Time-Series Study
Source: JMIR Public Health Surveill. 2024 Jul 23;10:e51883. doi: 10.2196/51883 (PMC11287102; doi:10.2196/51883)

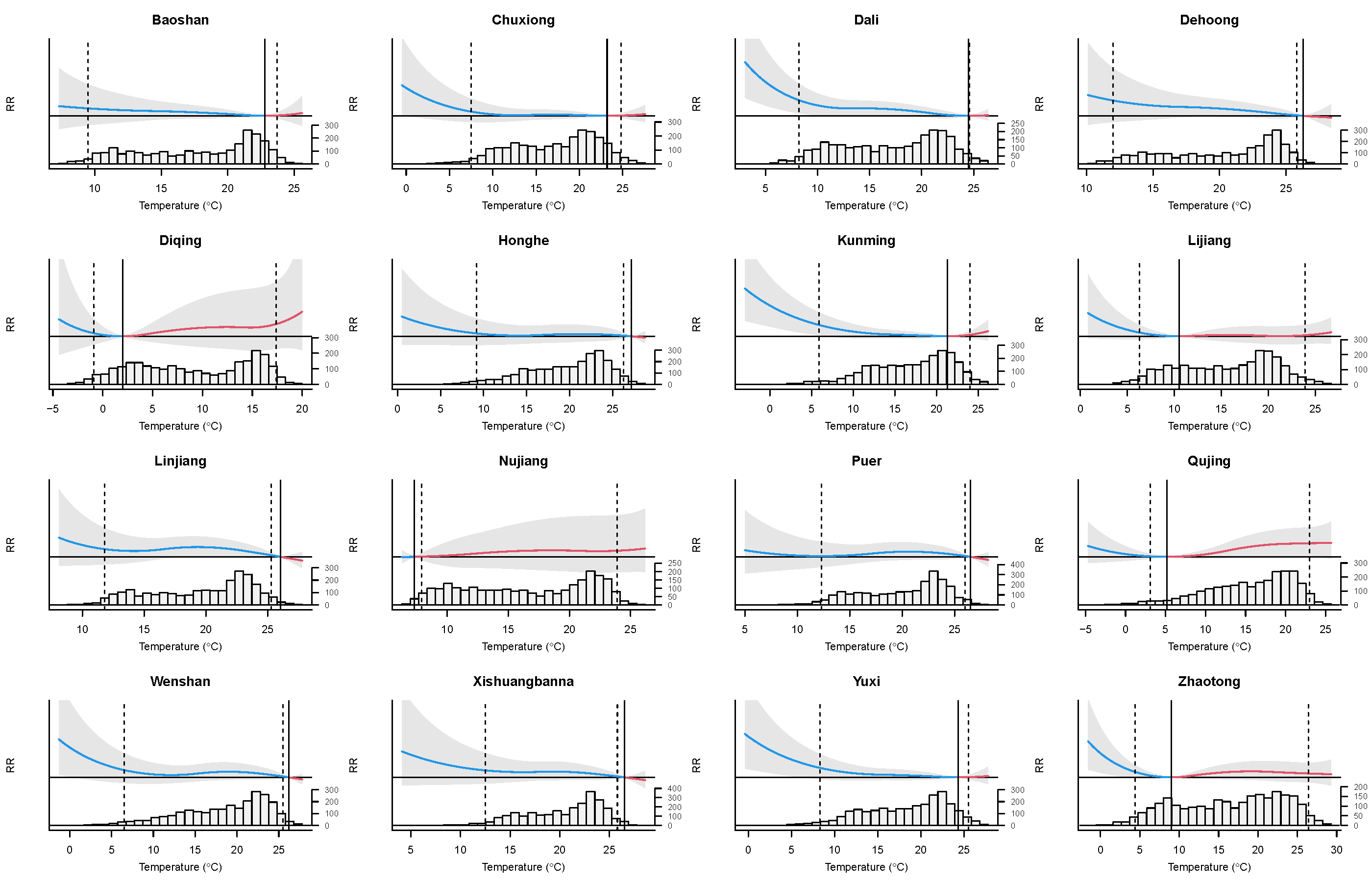

Supplement: Multimedia Appendix 3 [file publichealth-v10-e51883-s003.png]
