# Supplementary material for: Impact of Ambient Temperature on Mortality Burden and Spatial Heterogeneity in 16 Prefecture-Level Cities of a Low-Latitude Plateau Area in Yunnan Province: Time-Series Study
Source: JMIR Public Health Surveill. 2024 Jul 23;10:e51883. doi: 10.2196/51883 (PMC11287102; doi:10.2196/51883)
Supplement: Multimedia Appendix 4 [file publichealth-v10-e51883-s004.docx]

Table S3 Sensitivity analyses to calculate the estimated YLL with 95% empirical confidence interval attributable to temperature by changing maximum lag for mean temperature and degrees of freedom (*df*) for covariates

| **Model parameters** | Overall effect | $\text{Cold}^{a}$ effect | $\text{Heat}^{a}$ effect |
| --- | --- | --- | --- |
| Maximum lag time, days |  |  |  |
| 14 | 673.45 (324.83, 912.45) | 473.34 (243.33, 672.36) | 200.11 (-10.59, 365.91) |
| 28 | 810.02 (478.32, 1075.53) | 651.42 (399.59, 836.69) | 158.6 (-57.8, 338.3) |
| $\text{df}^{b}$ for time |  |  |  |
| 6 | 451.39 (209.2, 633.12) | 187.54 (35.22, 316.61) | 263.85 (74.42, 422.57) |
| 8 | 796.26 (426.65, 1099.4) | 369.3 (151.39, 539.66) | 426.96 (106.03, 688.79) |
| *df* for relative humidity |  |  |  |
| 2 | 643.49 (342.43, 894.1) | 403.21 (173.41, 607.84) | 240.27 (45.21, 401.09) |
| 4 | 655.18 (330.51, 896.67) | 407.03 (163.83, 611.05) | 248.16 (42.88, 408.38) |
| *df* for sunshine duration |  |  |  |
| 2 | 640.68 (313.36, 885.95) | 393.83 (152.9, 594.68) | 246.85 (41.06, 403.31) |
| 4 | 651.71 (349.27, 905.24) | 404.6 (160.91, 606.91) | 247.11 (47.9, 410.01) |
| *df* for average wind speed |  |  |  |
| 2 | 655.46 (331.84, 914.99) | 403.78 (175.65, 608.51) | 251.68 (51.02, 419.3) |
| 4 | 640.89 (320.82, 881.95) | 396.25 (162.41, 591.7) | 244.64 (26.14, 397.5) |
| *df* for atmospheric pressure |  |  |  |
| 2 | 646.54 (357.41, 873.23) | 391.62 (162.82, 588.17) | 254.93 (67.84, 415.09) |
| 4 | 647.22 (364.09, 890.09) | 392.99 (167.19, 588.66) | 254.24 (55.73, 416.37) |
| $\text{ }^{\text{a}}$Cold and heat were defined as those below and above the temperature with lowest YLL.  $\text{ }^{b}$*df*: degrees of freedom. | | | |

Table S4 Sensitivity analyses to calculate the attributable fraction (%) with 95% empirical confidence interval by changing maximum lag for mean temperature and degrees of freedom (*df*) for covariates

| **Model parameters** | Overall effect (%) | $\text{Cold}^{a}$ effect (%) | $\text{Heat}^{a}$ effect (%) |
| --- | --- | --- | --- |
| Maximum lag time, days |  |  |  |
| 14 | 7.71 (3.72, 10.44) | 5.42 (2.79, 7.7) | 2.29 (-0.12, 4.19) |
| 28 | 9.27 (5.47, 12.31) | 7.46 (4.57, 9.58) | 1.82 (-0.66, 3.87) |
| $\text{df}^{b}$for time |  |  |  |
| 6 | 5.17 (2.39, 7.25) | 2.15 (0.4, 3.62) | 3.02 (0.85, 4.84) |
| 8 | 9.11 (4.88, 12.58) | 4.23 (1.73, 6.18) | 4.89 (1.21, 7.88) |
| *df* for relative humidity |  |  |  |
| 2 | 7.37 (3.92, 10.23) | 4.62 (1.98, 6.96) | 2.75 (0.52, 4.59) |
| 4 | 7.5 (3.78, 10.26) | 4.66 (1.88, 6.99) | 2.84 (0.49, 4.67) |
| *df* for sunshine duration |  |  |  |
| 2 | 7.33 (3.59, 10.14) | 4.51 (1.75, 6.81) | 2.83 (0.47, 4.62) |
| 4 | 7.46 (4, 10.36) | 4.63 (1.84, 6.95) | 2.83 (0.55, 4.69) |
| *df* for average wind speed |  |  |  |
| 2 | 7.5 (3.8, 10.47) | 4.62 (2.01, 6.96) | 2.88 (0.58, 4.8) |
| 4 | 7.34 (3.67, 10.09) | 4.54 (1.86, 6.77) | 2.8 (0.3, 4.55) |
| *df* for atmospheric pressure |  |  |  |
| 2 | 7.4 (4.09, 9.99) | 4.48 (1.86, 6.73) | 2.92 (0.78, 4.75) |
| 4 | 7.41 (4.17, 10.19) | 4.5 (1.91, 6.74) | 2.91 (0.64, 4.77) |

$\text{ }^{\text{a}}$Cold and heat were defined as those below and above the temperature with lowest YLL.

$\text{ }^{b}$*df*: degrees of freedom.
